# Great Possibilities in Prospect for the Nursing Profession

**Published:** 1919-08-09

**Authors:** 


### MINISTERS OF HEALTH.

### Great Possibilities in Prospect for the Nursing Profession.

It would be both useful and interesting to discover, if it were possible, to what extent hospital matrons and committees have given consideration to the magnitude of the task with which the newly established Ministry of Health is confronted. The country may be compared with a block of polished timber in a garden of flowers. Everything is fair to the outward view; but, if the block is raised, behold the unclean clustering myriads of the underworld, scuttling from the sunlight. In the dark they live and breed and gnaw into the heart of the timber. People who inhabit wholesome dwellings, humble or otherwise, little realise that every day of the week marches a procession of funerals from the slums, and that if the names of the victims were chronicled in the Press, as were our war casualties, the death-roll would be better realised. Add to this the crippled and the degenerate who survive, and you have presented for contemplation the task that the nation has undertaken. Nobody has the opportunity of appreciating more accurately the colossal nature of the task and the urgent importance of reform than the hospital matron and the hospital managers and staffs. Everyday and all day their hands are fully occupied, for the sick poor throng their gates.

### A Great Opportunity.

I am not sure, however, that these valiant workers generally take any other than a purely local view of the situation, and far be it from me to suggest that it is their duty so to do. If a man's or a woman's time and energy are already fully taxed it seems an impertinence to suggest that they should be expected to bear extra burdens on their shoulders. And yet, at the same time, it is obvious that they must either do this, or, in the alternative, miss a great opportunity. We have in England to-day a gentleman described in Acts of Parliament as the Minister of Health—spelt with capital initials. We have also a vast army of health ministers, not spelt with capital initials, and not mentioned in Acts of Parliament—otherwise trained nurses. And the question I have to put is whether these are or are not to be entrusted with the reforms that are in contemplation. This question is of supreme importance, not only to the trained nurses, but to the nation. They are, without doubt, the natural custodians of the nation's health, but I doubt if this is realised or appreciated. So far as I am able to observe, it is not. Nurses are trained in excellent fashion to fulfil a particular and purely local task. But for duty off the beaten track there is no consideration. In other words, a trained nurse is not competent to discharge the duty of a public health officer, nor is she eligible for appointment.

### The Magic of Education.

In a recent article I dwelt in what I called the magic of education. Hospital committees and hospital matrons believe in this, but in a very limited degree. The local institution and the local requirements thereof mark the boundaries of their vision. They require probationers to discharge certain duties, and they educate them up to a certain point, that point being the immediate and pressing demands of the hospital in question. There they stop dead. It is a hand-to-mouth policy, and wholly incompatible with progress. If nursing is to be a profession in the full sense the whole system of training must be revised. The nurse-probationer must become a nurse-pupil, and when her course of training is complete she must emerge with a diploma that will enable her to take premier place in the national-health campaign in contemplation.

### Half-time Workers, Half-time Students.

If this is to become a *fait accompli* the first essential step must be a reduction of the hours of work for all probationers everywhere. They must be made half-time workers, and half-time students, and the total number of hours so employed must not exceed eight per diem. I do not like to use the word, but the old, and where unreformed, present, system is slavery. It would be a refinement of cruelty to demand more from probationers than they are now giving. I challenge any advocate of the present system to name a class of woman or man preparing for trade or professional life where the hours of duty are so long or so exacting. Take a bricklayer, a draper, a solicitor, a doctor. The apprentice or the student, as the case may be, has a king's time in comparison with that of the nurse-probationer, and in each of the cases named the worker is fully educated by the time the period of training is completed. In filling appointments under the Ministry of Health the trained nurse will be put into competition, and at a disadvantage with women not half her worth, simply because her educational training is reduced to the narrowest possible limits.

### The Position of the College.

Heaven be thanked, there is a College of Nursing, and within its ranks are women of wide outlook, who contemplate with confidence seeing the working nurse enjoying a career useful as well as remunerative. These pioneers have a terribly up-hill path to tread. They have open enemies to fight, which is comparatively simple, but, in addition, they have to surmount huge obstacles of tradition, and, worst of all, the apathy, not only of doctors, but of matrons—people who, indeed, believe that they are the allies of progress, but who are, in actual fact, the most conservative of autocrats. There are in prospect great possibilities for the nursing profession, but they are by no means certainties. The only hope of salvation lies in the democratic constitution of the College and its ardent and whole-hearted support by the rank and file.

IERNE.

